# Improved Skills for Predicting Sub‐Seasonal to Seasonal Global Sea Surface Temperature

**DOI:** 10.1002/advs.78271

**Published:** 2026-10-15

**Authors:** Longhao Wang, Xuanze Zhang, Ruby L. Leung, Francis H. S. Chiew, Amir AghaKouchak, Kairan Ying, Yongqiang Zhang

**Affiliations:** ^1^ Key Laboratory of Water Cycle and Related Land Surface Processes Institute of Geographic Sciences and Natural Resources Research Chinese Academy of Sciences Beijing China; ^2^ College of Resources and Environment University of Chinese Academy of Sciences Beijing China; ^3^ Pacific Northwest National Laboratory Richland Washington USA; ^4^ CSIRO Environment, Black Mountain Canberra ACT Australia; ^5^ Center for Hydrometeorology and Remote Sensing University of California Irvine California USA; ^6^ National Institute of Natural Hazards Ministry of Emergency Management of China Beijing China

**Keywords:** AI weather models, air‐sea interactions, deep learning, sea surface temperatures, sub‐seasonal to seasonal forecasting, weather forecasting

## Abstract

Accurate prediction of global sea surface temperature at sub‐seasonal to seasonal (S2S) timescales is critical for drought and flood forecasting, as well as for improving disaster preparedness in human society. Operational physics‐based numerical models are run to predict S2S sea surface temperature and corresponding climate indices, such as El Niño‐Southern Oscillation. However, these models are hampered by computational inefficiencies, rapidly dissipating ocean‐atmosphere initial conditions, and significant uncertainty and biases. Here, we introduce a novel spatiotemporal deep learning neural network to model the nonlinear and complex coupled atmosphere‐ocean weather systems. This model, named as CAS‐Canglong, incorporates climatic and temporal features and employs a self‐attention mechanism to enhance the prediction of global S2S sea surface temperature pattern. Compared to the physics‐based models, it shows higher computational efficiency and more predictive capability, improving one to three months sea surface temperature predictive skill by 55.6 ± 14.1% to 63.4 ± 15.5% in six ocean regions with dominant influence on S2S variability over land. This achievement underscores the huge potential of deep learning for largely improving forecasting skills at the S2S timescales over land.

## Graphical abstract

Addressing the critical Sub‐seasonal to Seasonal predictability gap, this study presents CAS‐Canglong, a novel deep learning framework. By leveraging 3D Swin‐Transformers to capture long‐range ocean interactions, the model demonstrates superior skill over traditional GCMs. With an unprecedented ENSO prediction accuracy, it offers robust support for global disaster preparedness and climate resilience.

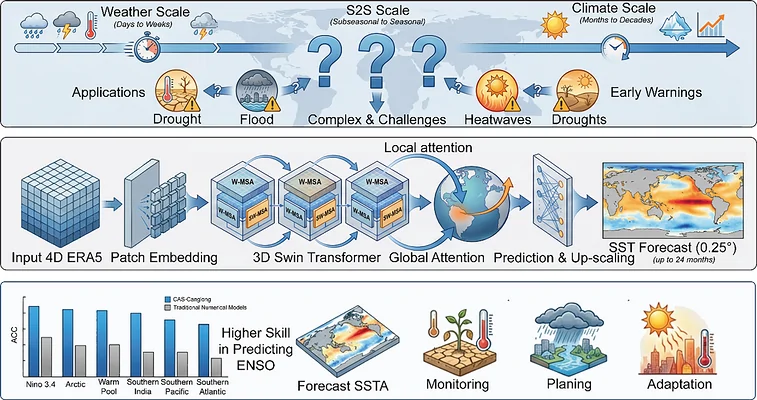

## 1 Introduction

At timescales ranging from two weeks to a few months, sub‐seasonal to seasonal (S2S) prediction is a major challenge at the weather‐climate transition [1, 2]. Despite steady progress in the last two decades, prediction skill at the S2S scale remains very low [3, 4, 5, 6]. Accurate S2S prediction of global sea surface temperature (SST), a key initial and boundary condition for numerical weather and climate prediction models, is crucial for improving the predictive skill of medium‐to‐long term weather forecasting. Global SST anomaly and associated climate variability modes exert a significant influence on extreme events through their modulation of atmospheric circulations [7]. For example, it is well‐known that the El Niño‐Southern Oscillation (ENSO) featuring tropical SST anomaly in the tropical Pacific Ocean plays a central role in seasonal to annual regional climate prediction [8, 9, 10]. Consequently, S2S prediction of ENSO events can provide early warning of extreme events over ENSO‐influenced regions with lead times ranging from several weeks to a few months [11, 12]. Extreme weather and climate events, including heat waves, droughts, floods, and cyclones, are becoming more frequent and severe [13, 14, 15], causing serious loss of life and economic damage. Developing robust S2S prediction methods is critical for enhancing early warnings, assessing risks, and supporting disaster preparedness.

Numerical modelling has been widely used by international major forecasting centres for predicting S2S variations of global SST [16, 17]. However, accurately predicting the S2S SST variability using general circulation models (GCMs) remains a great challenge, particularly for extra‐tropical SST because GCMs exhibit large biases owing to inadequate representation of sea‐air interactions and atmospheric processes connected to large‐scale atmospheric circulations [18]. Utilizing partial differential equations and physics parameterizations to represent the coupled ocean‐atmosphere system, the large computational requirements of GCMs have limited their spatial resolution and ensemble size, contributing to their relatively low S2S SST prediction skill [19, 20]. Since atmospheric forecasts generally lose skill beyond two weeks due to the chaotic theory of the atmosphere, uncertainty in predicting the global SST pattern becomes a key factor limiting predictability at the S2S timescales [21, 22, 23].

Unlike physics‐based modelling methods, data‐driven deep learning (DL) models leveraging high‐dimensional and long‐time‐scale multivariate data can be used to predict and explain SST by learning patterns and intrinsic physical relationships from historical observations [24]. Previous studies have used the Long Short‐Term Memory (LSTM) network in SST prediction [1, 25, 26, 27]. Different variants of the LSTM methods have become the de facto standard due to their ability to capture contextual information and suitability for time‐series SST modelling. However, LSTMs primarily focus on local pixel data, limiting their application in high‐dimensional multivariate modelling and failing to capture the complex dynamics of ocean‐atmosphere interactions [28]. This limitation renders them less effective for sub‐seasonal weather prediction and detecting extreme weather anomalies.

Recent advances in DL‐based artificial intelligence (AI) models have spurred their applications in the Earth science community, particularly in short to medium‐range global weather forecasting [29, 30, 31, 32], and seasonal‐to‐annual ENSO prediction [33, 34]. Recent specialized deep learning models have achieved strong performance in monthly SST and ENSO forecasting by explicitly modeling multivariate air–sea coupling dynamics [35], learning global spatiotemporal teleconnections [36], and integrating causal inference for lead‐dependent predictions [37]. Incorporating these innovative architectures provides critical insights for advancing data‐driven climate forecasting. These AI‐driven weather models collectively contribute to WeatherBench [38], a framework designed for evaluating and comparing data‐driven and traditional numerical weather prediction models. It provides publicly available models and baseline datasets. However, these analyses are predominantly focused on the weather scale (i.e., less than two weeks), with very few developments and predictions dedicated to AI models at the S2S scale. To date, there have been no reports using DL models explicitly predicting S2S global SST. While current AI models are not specifically designed for S2S global SST prediction, some of them may have potential to predict SST at the S2S scale.

Here we develop an AI‐based model using a self‐attention‐based Transformer architecture, named CAS‐Canglong that aims for improving prediction of SST at the S2S timescales. Our implementation of the three‐dimensional (3D) Transformer establishes multivariate relationships across space and time. In brief, CAS‐Canglong ingests the preceding 16 months of eight ERA5 fields—SST, sea‐level pressure, zonal and meridional wind speed, 850‐ and 500‐hPa geopotential heights, surface latent heat flux, and net solar radiation—on a 0.25° × 0.25° global grid and issues monthly SST forecasts at lead times of 1–3 months as the primary evaluation target, with capability extending up to 16 months. Specifically, we use 51 years (1959‐2009) of reanalyses data for eight key oceanic and atmospheric variables to train the CAS‐Canglong, while validating and testing the model in 2009–2015 and 2016–2022, respectively. CAS‐Canglong demonstrates its superior and robust capabilities in predicting SST seasonal cycles, anomalies, and SST‐based climate indices in both hindcast and near‐real‐time forecasting modes, as detailed in the Result Section.

## 2 Results

### 2.1 Hindcast Comparisons

To quantitatively assess the performance of our AI‐model's SST predictions, we compared CAS‐Canglong with the numerical models based predictions obtained from ECMWF S2S model ensemble [39] and NMME North American multi‐model ensemble [40] for the overlapping period 2016–2020. CAS‐Canglong has a spatial resolution of 0.25°×0.25°, which is the finest available and comparable to that of the physics‐based models. The spatial resolution of our model was remapped to match the resolution of the physics‐based models, most of which have a spatial resolution of 1°×1°. The prediction lead time is set to 3 months for the assessment, and we compare the spatial pattern Anomaly Correlation Coefficient (ACC) and Anomaly Root Mean Square Error (ARMSE) of SST predictions, the two metrics for evaluating spatial performance (see Methods), in several key regions and associated climate indices. The predictive skill for SST across different regions is summarised in Figure 1, indicating that CAS‐Canglong gives better SST predictions for one, two and three months lead time compared to the numerical models. From Figure 1, CAS‐Canglong shows more accurate and reliable predictions than numerical models from one‐ to three‐month scales (see also Figure S1). CAS‐Canglong gets higher ACC scores in five of the six regions (except for the Southern Indian Ocean).

**FIGURE 1:**
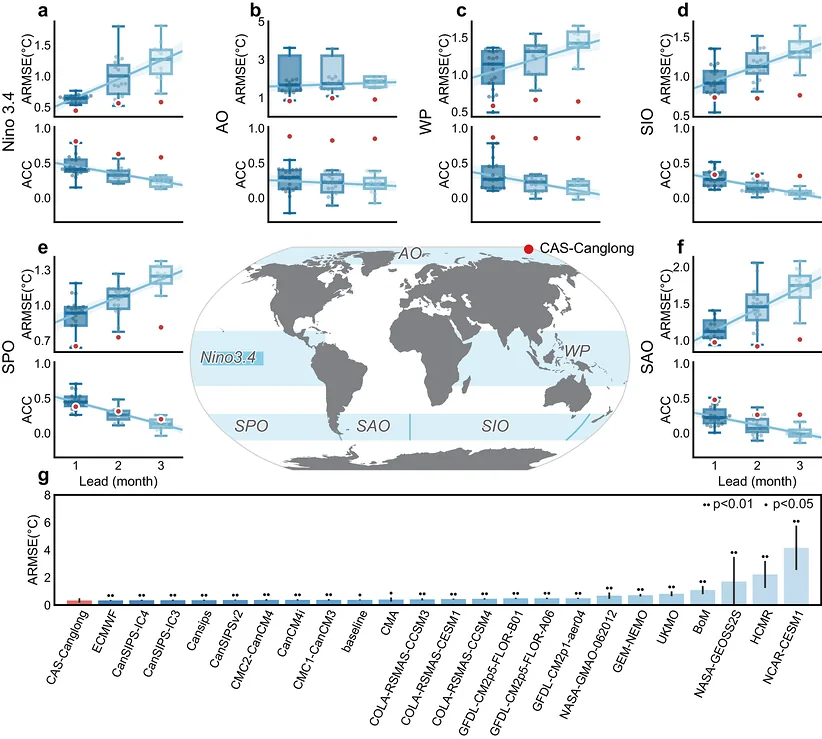
Predictive skill assessment for the deep learning‐based S2S model (CAS‐Canglong) and the state‐of‐the‐art GCM S2S models. The one to three‐month lead predictions of SST from CAS‐Canglong are compared to those from 22 state‐of‐the‐art NWP models. The pattern anomaly correlation skill (ACC) and latitude‐weighted ARMSE are summarized in box plot, by key SST regions and associated SST climate indices. For a robust comparison, the calculated correlation skill and ARMSE (in°C) are based on the mean values from 2016 to 2020, which represents an overlap between CAS‐Canglong's testing period and availability of the physical‐based data. The red dots represent the CAS‐Canglong. The boxplot shows the ACC and ARMSE distribution of different models. Each sub‐boxplot shows the lower whisker (Q1‐1.5IQR), lower quartile (Q1), median, upper quartile (Q3), and upper whisker (Q3+1.5IQR), where the IQR = Q3‐Q1. (a) Niño 3.4 region (170°W‐120°W, 5°S‐5°N). (b) Arctic Oscillation region (75°N‐90°N). (c) Tropical Warming Pool (WP) region (35°E‐75°W, 15°S‐15°N). (d) Southern Indian Ocean (SIO) region (20°E‐ 160°E, 40°S‐60°S). (e) Southern Pacific Ocean (SPO) region (160°E‐70°W, 40°S‐60°S). (f) Southern Atlantic Ocean (SAO) region (70°W‐20°E, 40°S‐60°S). (g) Global mean ARMSE (in°C) of the CAS‐Canglong and the numerical S2S models compared with the ERA5 ground truth. In (g) all predictions are for one‐month lead of global SST from 2016 to 2020. The shaded areas denote the fifth to 95th percentile of ensemble model prediction. The error bars represent the mean error ± one standard deviation. The persistence baseline assumes that the SSTA at a given time remains unchanged for future lead times. Asterisks * and ** indicate a significant difference between CAS‐Canglong and the other GCM models at the p < 0.10 and p < 0.05 levels, respectively.

Comparing the prediction error with all the GCMs, CAS‐Canglong achieves the lowest error, surpassing all the numerical models. A one‐sided test with Benjamini–Hochberg false‐discovery‐rate correction showed that CAS‐Canglong had significantly lower global ARMSE than 21 of the 22 NWP models (p < 0.01). The improvement (reduction of ARMSE) at the S2S scale is 56.7 ± 9.4%, 56.6 ± 20.6%, 56.3 ± 8.5%, 57.6 ± 11.5%, 63.4 ± 15.5%, and 55.6 ± 14.1%, for the regions of Niño 3.4, Arctic Oscillation, Warm Pool, Southern Atlantic Ocean, Southern Indian Ocean, and Southern Pacific Ocean, respectively. For a robust comparison, we also evaluated our SST predictions against the Hadley Centre Sea Surface Temperature observational dataset. Using the same evaluation method, CAS‐Canglong again achieved outstanding performance for all the regions investigated (Figure S2). Notably, our model generates SST predictions for lead times ranging from 1 to 16 months, with much lower prediction errors. Additionally, CAS‐Canglong model nearly matches the current state‐of‐the‐art SST prediction models, producing predictions highly consistent with those of the ECMWF forecast (Figure S3). Based on the outstanding overall performance of CAS‐Canglong, we focus on its results in key SST regions compared with the multi‐models in the following sections. CAS‐Canglong predicted the global SST anomalies (SSTA) for a weak La Niña event in January 2021, as shown in Figure 2. In addition to demonstrating high global SST prediction skill, CAS‐Canglong was able to accurately capture the global SSTA distribution (Figure S4), even for relatively weak ENSO events (Figures S5–S6). The prediction skill for SST anomalies at a lead time of one month reached 0.62, and strong correlations for SST anomalies were maintained for lead times of up to 10 months. However, the correlation rapidly decayed beyond a 12‐month lead time. The evaluation also provides some evidence of skill in predicting marine heatwaves (Figures S7–S9).

**FIGURE 2:**
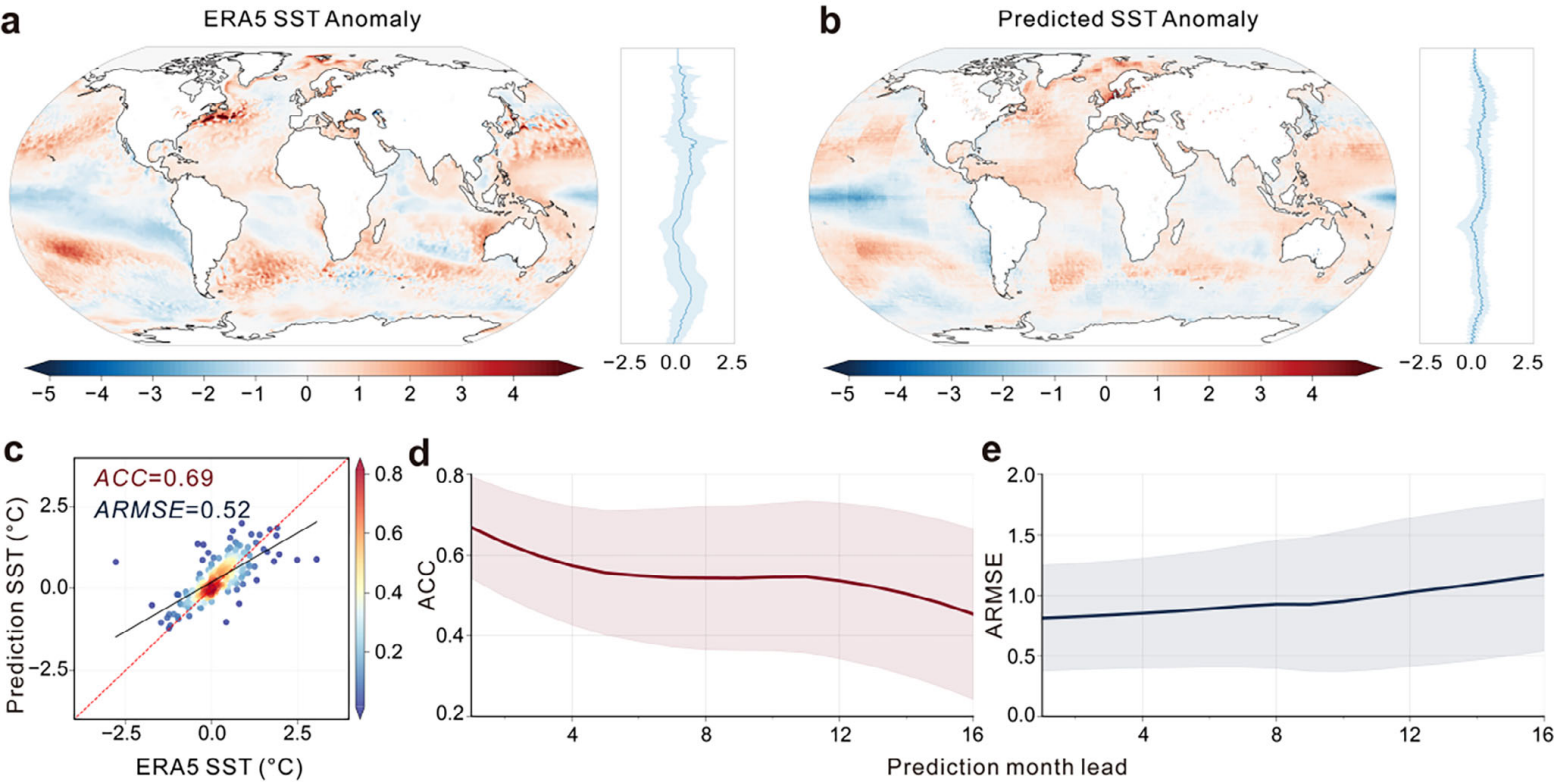
Visualization of global SST anomalies predictions and validation accuracy. (a) The ERA5 ground truth. (b) The 1‐month advance prediction of SST anomalies. An example spatial map is provided for the 2020 La Niña event during the test period, highlighting the latitudinal distribution of SST. (c) This scatter plot compares the ERA5 ground truth SST anomalies (x‐axis) with the predicted SST anomalies (y‐axis) in one month advance globally. (d, e) Mean values of pattern ACC (red) and ARMSE (blue) over the test period, plotted against prediction lead months for global SST anomalies predictions. The shaded areas represent ± one standard deviation, illustrating the variability in the data.

### 2.2 Hindcast in Major Regions

Here, we focus on the six SST regions and associated indices (see Methods), including the regions of Niño 3.4, Arctic Oscillation (AO), warm pool (WP), Southern Atlantic Ocean (SAO), Southern Indian Ocean (SIO), and Southern Pacific Ocean (SPO). Figure 3 shows the 1‐month hindcast SSTA or SST‐based climatic indices for these regions versus the ERA5 ground truth, providing insights into the capability of predicting the spatial distribution of sea surface temperature anomalies one month in advance. CAS‐Canglong shows strong forecast skills in all the regions. In tropical regions, specifically the Indo‐Pacific warm pool area with sea surface temperatures (SSTs) exceeding 28°C, the model's performance is shown during the single‐month of January 2021 with a weak La Niña event (Figure 3) and across the multi‐year mean warm pool area (Figure S10). The predicted warm pool area is 4.76×107 km^2^, compared to the ERA5 of 4.27 × 10^7^ km^2^, resulting in a low error of 10.3% (Figure 3c). For the temperate Southern Oceans, including the SIO, SPO, and SAO (Figures 1d‐f and 3d–f), the spatial distribution of predicted SSTA is highly consistent with the ERA5. For the three‐month lead predictions, the model shows superior performance for the SIO, SPO, and SAO with a mean spatial correlation value of 0.41 for all Southern Ocean regions (Figure 3d–f). These results indicate our model's strong performance not only in tropical but also in temperate and high latitude regions.

**FIGURE 3:**
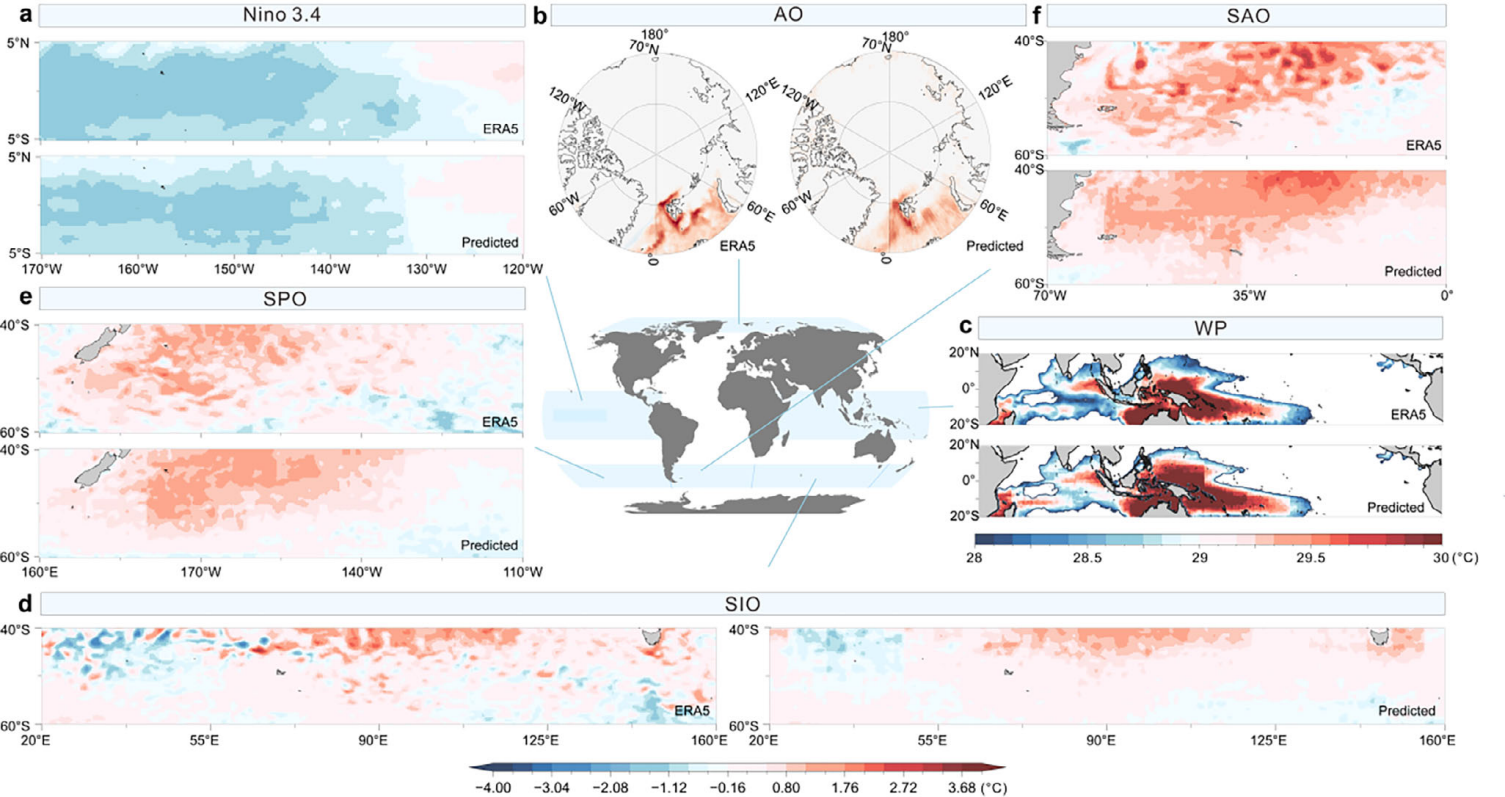
Comparison of spatial pattern between observed SSTA and Canglong‐predicted SSTA in selected six global regions. An example of a spatial map in January 2021 with a weak La Niña event (same as Figure 2). For each panel of a specific SST region, the spatial maps at the top or on the left show the one‐month lead predictions of ERA5 ground truth, with the corresponding spatial maps at the bottom or on the right showing the CAS‐Canglong. Corresponding with Figure 1, the regions are for (a) Niño 3.4, (b) AO, (c) WP, (d) SIO, (e) SPO, and (f) SAO.

We now focus on the entire test period from May 2016 to December 2022 for the SST regions and related climate indices. The CAS‐Canglong time series and the ground truth for the SST and related climate indices are presented in Figure 4, with predictions categorized by S2S lead times of 1 to 3 months. We selected the most important SST index at S2S timescales, the ENSO‐related Niño index, to elucidate the model's predictive capabilities. Our analysis emphasizes the accuracy in forecasting El Niño and La Niña events across the entire test dataset. The temporal evolution of the predicted Niño 3.4 index closely aligns with the ERA5 for one‐ to three‐month lead predictions, achieving correlation values of 0.90, 0.89, and 0.66, respectively (Figure 4a). Extended evaluation of Niño 3.4 Index compared with the persistence skill and predictions from the traditional multiple linear regression models could be found in Figure S11. To evaluate the reliance on ocean memory, the predictions were additionally benchmarked against persistence and traditional linear regression models up to a 12‐month lead (Figures S12‐S13). The model accurately captures all the ENSO events during 2016–2022, achieving an overall hit rate of 75% for three months in advance. In the tropical region, our model demonstrates outstanding performance not only in simulating the Niño 3.4 index but also the Warm Pool area. As shown in Figure 4c, the model accurately captures the variations of the dynamic Warm Pool area, with correlation skill > 0.7 for three months in advance. In the Southern Ocean, the model shows its superior performance, with prediction correlations reaching 0.9 and 0.7 for one‐month and three‐month lead time, respectively. The model well captures the seasonal variations in SST of the Southern Ocean. For the AO in polar regions, where traditional NWP models often struggle to simulate and predict, our model also exhibits remarkable performance, with SST prediction correlations reaching 0.9 and 0.6 for one‐month and three‐month lead times, respectively (Figure 4b). The SSTA prediction skill also remains robust across these regions, with correlation coefficients mostly exceeding 0.5 (Figure S14).

**FIGURE 4:**
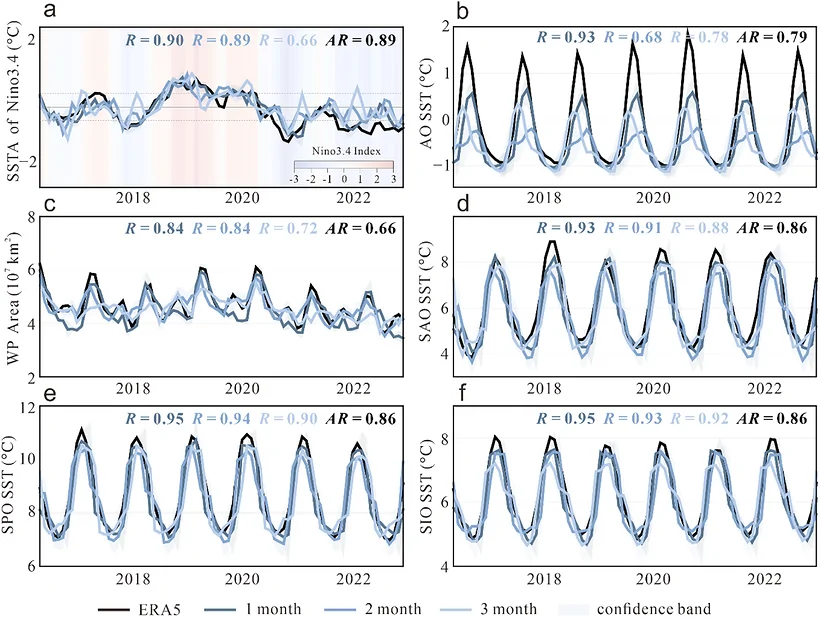
Time series of SST predictions and climate indices in six major regions for the period 2016–2022. Predictions are conducted every month from 2016 to 2022, and the results are merged to generate a single time series. The black line represents the ERA5 ground truth, while the blue lines show the model predictions for one to three months lead time, with colors ranging from dark to light blue corresponding to one, two, and three months, respectively. The background shading of the time series is overlaid with ± one standard deviation. The regions correspond with Figure 1 & Figure 3: (a) Niño 3.4, (b) AO, (c) WP, (d) SAO, (e) SPO, and (f) SIO. The Niño index (shaded) is marked along the x‐axis in (a). AR denotes the one month lag autocorrelation coefficient of the observed index anomalies over the test period.

### 2.3 Near Real‐Time Forecasting

To evaluate our model's real‐time forecasting performance, Figure 5 presents the predicted SST anomalies for the Niño 3.4 region in 2024, compared against data for March to June 2024 obtained from the latest ERA5 observations. Availability of the latter allows us to forecast the subsequent months (July‐December). The ensemble models’ predictions from the International Research Institute (IRI) are also shown in Figure 5. In the first three months of the year 2024, a typical El Niño event with positive SST anomaly is observed, with a potential shift toward a La Niña event with negative SST anomaly in the latter half of the year. CAS‐Canglong successfully predicted this transition, with higher predictive accuracy than the ECMWF S2S model. Figure 5 also demonstrates that during the El Niño months, our model achieves reliable results, nearly matching the ensemble mean of the current state‐of‐the‐art dynamical models or statistical models. CAS‐Canglong captured the observed earlier transition time point (during AMJ) than both the ensemble predictions of dynamical and statistical models. These results validate our model's capability for S2S scale predictions in near real time, offering both faster inference speeds and enhanced accuracy.

**FIGURE 5:**
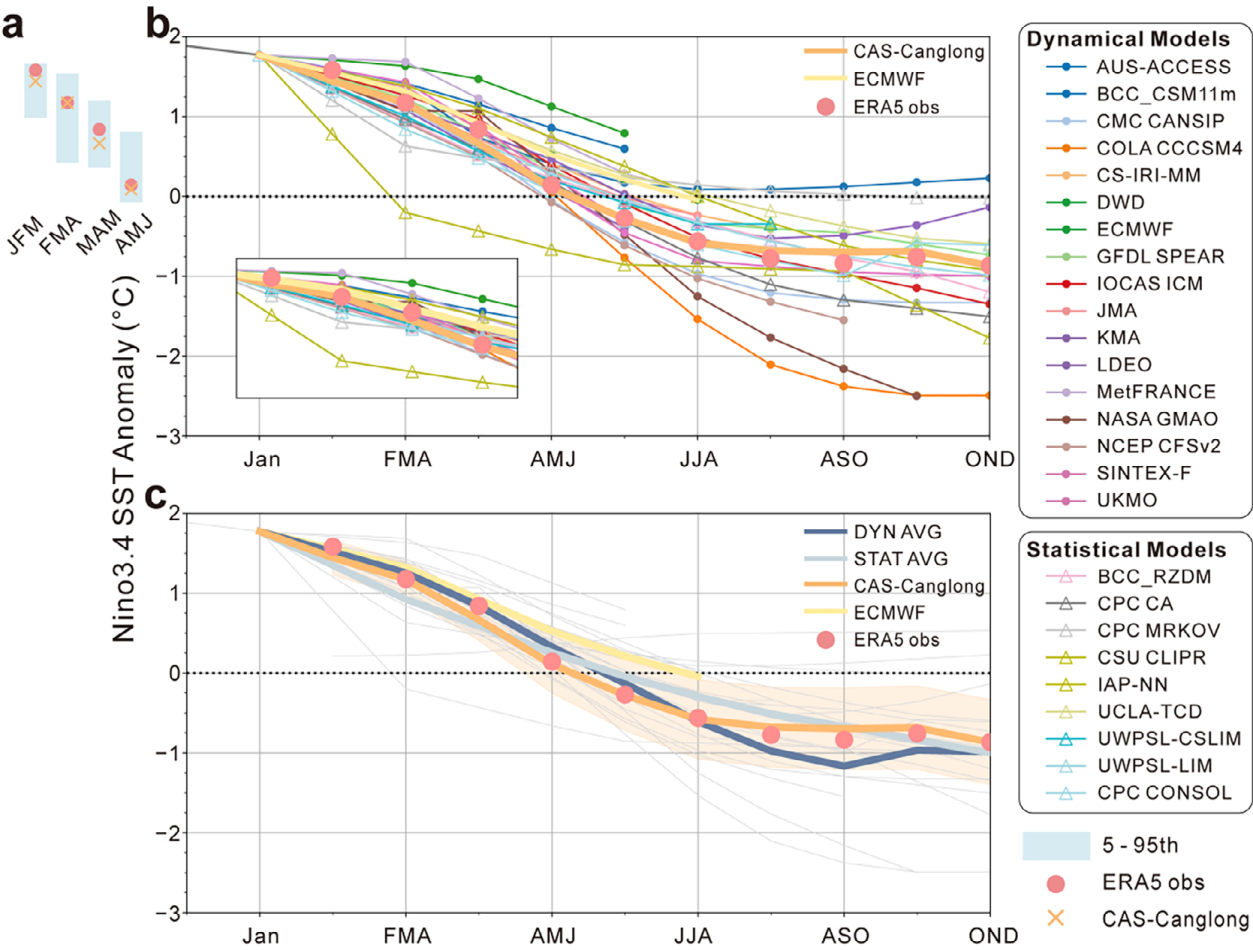
Near real‐time predictions for the Niño 3.4 SST anomalies since January 2024. These ensemble model datasets were obtained in January 2024 from the International Research Institute (IRI), with the ERA5 ground truth updated in real‐time. (a) The magnified section of the graph shows the data from January to June 2024. The blue boxes indicate the 95th percentile of the ensemble predictions from all dynamical and statistical models, yellow crosses represent CAS‐Canglong's predictions, and red dots mark the ERA5 ground truth, highlighting CAS‐Canglong's prediction within the ensemble predictions of all other models and, its proximity to the true values. (b) Multi‐model predictions for the Niño 3.4 SST anomalies are displayed, including our model's predictions alongside the ERA5 ground truth (red dot). (c) Other model lines are faded out and, summarized as IRI statistical ensemble‐averaged predictions (dark blue) and dynamical ensemble‐averaged forecasts (blue). Lines for the ECMWF S2S model and the CAS‐Canglong are highlighted for comparison.

## 3 Discussion

Forecasting at the S2S scale across global land is critical for early warning and emergency preparedness of major extreme weather and climate events such as heatwave and droughts. The seasonal variability of SST on the S2S scale has profound impacts on atmospheric variability over land, so accurate prediction of SST is necessary for predicting extreme events that affect terrestrial ecosystems and human societies [41]. However, traditional physics‐based dynamical models still have low predictability on the S2S scale [42]. The major challenge is that the S2S time range is much longer than the memory of atmospheric processes but is too short for the sea surface variability to have a strong influence relative to the variability of atmospheric circulation [2, 23]. Global sea surface temperatures, particularly the tropical SSTs over the Indo‐Pacific Warm Pool region and the eastern tropical Pacific Ocean (e.g., the Niño 3.4 region), exert the dominant roles in global subseasonal‐to‐seasonal variability of hydroclimate extremes by releasing huge amounts of water vapour and latent heat to the atmosphere which modulate atmospheric circulations and the spatiotemporal distributions of global land precipitation [43]. SST is a crucial factor in predicting precipitation and extreme weather events and assessing extreme weather risks [43]. Furthermore, ENSO events on the S2S scale strongly impact extreme precipitation and droughts, with outsized impacts in densely populated regions [44]. Therefore, enhancing the accuracy of SST predictions on the S2S timescales could substantially improve forecasting of extreme events which are increasingly affecting humans and societies due to anthropogenic global warming. We therefore focus predictions on 1–3‐month leads, a critical S2S window often deemed a “predictability desert” for deterministic forecasting as atmospheric memory rapidly dissipates. Beyond three months, operational forecasting generally shifts from deterministic quantitative accuracy toward qualitative anomaly tendencies. Nevertheless, to meet diverse operational needs, CAS‐Canglong readily provides extended predictions at longer horizons (4–6 and 6–12 months).

Recent advances in data‐driven, attention mechanism‐based deep learning models offer a promising approach for weather forecasting and seasonal‐to‐annual ENSO predictions [33, 45]. Inspired by the recent successes of the Transformer model in computer vision [46, 47], such as the Sora model's ability to predict video sequences up to one minute long [48], we recognized the potential for similar applications in weather forecasting and climate prediction for enhancing long‐term predictive capabilities in Earth sciences [49]. Recently, widely tested AI models, such as Pangu [29], GraphCast [30], NeuralGCM [50], and GenCast [51], have demonstrated greater potential than traditional numerical models in simulating medium‐range weather. These models have made important contributions to climate modeling and exhibit strong predictive skills. However, at the S2S timescales, very few AI models have been tested. For comparison, we selected two additional AI models: 3D‐CNN [34] and 3D‐Geoformer [33] (Figures S15 and S16) alongside the ARIMA model as another baseline (Figure S17). It is worth noting that sophisticated models, such as 3D‐CNN, have not consistently outperformed some traditional numerical methods in terms of accuracy. In traditional weather models, iterative forecasting is common due to its reliance on accurate initial conditions. However, at the S2S scale, predictions are less sensitive to initial conditions, making iterative forecasting less effective.

Consequently, we developed CAS‐Canglong utilizing the Swin‐Transformer architecture and unique self‐attention mechanisms to advance long‐term modeling and prediction capabilities (Figure S24). CAS‐Canglong model shows clear advantages over other AI models in the following aspects: (1) directly producing global SST forecasts at multiple leads without iterative roll‐out, whereas several existing AI models are index‐oriented, limited to shorter horizons, or rely on iterative forecasting, which may lead to increasingly smoothed predictions at longer leads; (2) achieving better performance across most evaluated index‐based and ensemble skill metrics, and exhibiting the potential to improve S2S predictions of weather and climate anomalies; and (3) maintaining superior predictive skill against baseline deep learning models at multi‐month leads, whereas most existing machine‐learning models predominantly target daily weather‐scale forecasts rather than sub‐seasonal to seasonal SST evolution. Therefore, CAS‐Canglong model represents a breakthrough, successfully simulating and predicting global SST on sub‐seasonal to seasonal scales using deep learning, building upon the potential demonstrated by other recently released DL models.

CAS‐Canglong may enable early warning of extreme events by effectively predicting SST on sub‐seasonal to seasonal scales, capturing both the seasonal cycles and anomalies in sea surface temperatures. Our model aims to capture large‐scale SST variations by using raw SSTs (rather than anomalies) as both input and output, and the loss function is also primarily based on SST reconstruction. This design likely enhances the model's ability to capture seasonal signals embedded in the raw SST data [34]. Furthermore, we found that ARMSE values stabilize beyond an 8‐month lead, primarily because deep learning models capture statistical patterns rather than fully replicating physical dynamics, which provides baseline predictability even as lead times increase. Another reason is the model's ability to capture seasonal cycles and large‐scale patterns, which help stabilize errors. However, regional anomaly forecasts exhibit a phase‐dependent bias, underestimating positive peaks while overestimating negative troughs. This pattern indicates regression toward the climatological mean, a typical behavior when minimizing mean squared error over finite data. In contrast, many mainstream SST or ENSO prediction models, use SSTA as input. By removing the seasonal cycle, these models primarily focus on interannual variability and tend to ignore seasonality. The predictive prowess of our model has been demonstrated, not only for ENSO events but also for other modes of variability such as the Tropical Warm Pool area, AO, PDO, and the Southern Ocean SST anomalies, demonstrating a profound ability to model the air‐sea interactions processes. This understanding enables our model to accurately forecast extreme sea temperature anomalies of global key SST regions such as ENSO. Additionally, our model may have the ability to further predict ENSO‐affected land regions such as western North America and the Amazon. Our model achieves a 75% accuracy hit rate in predicting ENSO events three months in advance. Our model has noticeably better skills than most ECMWF S2S models and the ensemble‐averaged numerical model, showing great potential. Beyond operational forecasting, the high computational efficiency and spatiotemporal fidelity of CAS‐Canglong allow us to systematically apply physically constrained perturbations to trace lead‐dependent error growth and identify sensitive regions for ocean variability modes in the future work, aligning with the “AI for science” predictability framework [36, 52]. Extending CAS‐Canglong from predictive modeling to such process‐level predictability analyses represents a key direction for our future work. The current model also has some limitations and uncertainties. First, while the model is capable of generating ensemble forecasts, the uncertainty remains considerable, and it cannot be regarded as a truly robust ensemble system. The ensemble forecasting was implemented by introducing random perturbations, following the methodology of Pangu‐Weather, where Perlin noise was added to the initial conditions to create perturbations [29]. This approach allowed us to produce 21 ensemble members for each forecast. The extra skill and ensemble forecast skill metrics in Figures S18‐S20 indicate that our ensemble forecast performance ranks second among all ensemble models, rather than being the best. While random Perlin‐noise perturbations provide computationally efficient ensemble diversity, they lack state conditioning and physical constraints, potentially underestimating state‐dependent forecast uncertainty. To improve ensemble calibration and physical consistency, future work will explore advanced perturbation strategies, including dynamically targeted growing‐type perturbations and physics‐informed ensemble generation [53]. Second, the Swin‐Transformer architecture also shows some uncertainties in estimating SST‐related indices. Although the model performs well in simulating SST, artifacts may appear when calculating SST‐related indices or SSTA (e.g., as shown in Figure 3d). Moreover, extreme phases of the high‐latitude AO SST index remain difficult to capture beyond short lead times due to its weak oceanic memory and strong atmospheric noise. High‐latitude predictability is heavily dictated by atmospheric initial conditions and stratospheric processes, which present inherent constraints for subseasonal DL models [54, 55]. Similarly, SIO prediction skill is limited by SAM‐driven wind forcing, air–sea heat exchanges, and eddy‐rich ACC frontal meanders, where subtle spatial or phase misalignments penalize correlation skill (Figure S21). The root cause lies in the architecture of traditional 3D‐Transformer‐based models. Specifically, this issue can be attributed to the block embedding and recovery operations inherent in traditional AI models. The information loss caused by this deep learning structure is minimal and generally acceptable. As demonstrated by the Power Spectral Density analysis in Figure S22, the information loss is shown to be very limited. Finally, the deep learning model lacks explicit physical interpretability, though methods like SHAP offer some insight into its learned spatiotemporal features (see Figure S23).

In summary, we developed a novel deep learning model, CAS‐Canglong, for predicting global sea surface temperature at each 0.25°×0.25° grid cell. Our validations and predictions demonstrate CAS‐Canglong's outstanding capability for predicting S2S SST changes. This model can be directly applied to other sub‐seasonal Earth system weather prediction tasks, positioning it potentially to become a generalizable standard deep learning model in geoscience. As SST serves as the foundation for S2S weather forecasts over land, this work lays the groundwork for land‐based S2S weather predictions, such as precipitation, and has important implications for achieving skillful S2S forecasting in the future.

## 4 Experimental Section

### 4.1 Data

We use the monthly aggregated ERA5 reanalysis data for atmospheric and oceanic variables for this study. The ERA5 dataset [56] represents the fifth‐generation ECMWF atmospheric reanalysis of the global weather and climate, providing hourly reanalysis data spanning the past 60 years. As a cutting‐edge generation of reanalysis data, ERA5 is recognized for its high quality by blending numerical models with reanalysis data using numerical assimilation methods. This approach has been used for numerous applications including global earth surface predictions.

We retained the highest resolution available from the ERA5 dataset, specifically 0.25°×0.25° at the Earth's surface, comprising a total of 1,440×721 grids for each variable at each time step. For predicting SST, we followed widely accepted practices with dynamical underpinning [32, 33], selecting single‐level variables including sea level pressure, zonal and meridional wind speed and geopotential heights at 850‐hPa and 500‐hPa, surface latent heat flux, and net solar radiation as predictors. We adhered to a training, validation, and testing dataset division of 8:1:1. Specifically, the model was trained using monthly data from 1959 to August 2009, validated with the data from September 2009 to April 2016, and tested with data from May 2016 to December 2022. During the training phase, we monitored the performance on both the training and validation sets to avoid overfitting. Subsequently, we evaluated the model on an independent test set.

Additionally, we also obtained SST data from other observations and modelled. In addition to the ERA5 SST data, the Hadley Centre Sea‐Surface Temperature (HadSST) dataset is also used for model comparison as observations. The HadSST dataset provides gridded monthly SST data from 1850 to the present. It fully incorporates in situ measurements from ships and drifting buoys and is considered a high‐quality, standard SST dataset [57]. Additionally, we utilized the European Centre for Medium‐Range Weather Forecasts (ECMWF) S2S dataset and the North American Multi‐Model Ensemble (NMME) S2S SST for comparison with CAS‐Canglong. The NMME encompasses both real‐time, initialized predictions and a comprehensive research database, archiving both retrospective and real‐time forecasts. For comparison, we employed NMME ensemble models that provide SST forecasts, including CanCM4i, Cansips, CanSIPS‐IC3, CanSIPS‐IC4, CanSIPSv2, CMC1‐CanCM3, CMC2‐CanCM4, COLA‐RSMAS‐CCSM3, COLA‐RSMAS‐CCSM4, COLA‐RSMAS‐CESM1, ECMWF, GEM‐NEMO, GFDL‐CM2p1‐aer04, GFDL‐CM2p5‐FLOR‐A06, GFDL‐CM2p5‐FLOR‐B01, NASA‐GEOSS2S, NASA‐GMAO‐062012, and NCAR‐CESM1. The ECMWF S2S project and database include forecasts and reforecasts from both operational and research centers. We selected datasets with prediction lead times exceeding one month for comparison, including those from ECMWF, UKMO, HCMR, BoM, and CMA.

### 4.2 CAS‐Canglong Model Development

Inspired by the successful applications of Transformer models [29, 45] in spatiotemporal predictions and the unique challenges posed by SST‐related ocean‐atmosphere coupling and time‐series modeling, we developed a computationally efficient Transformer‐based model for SST predictions, named as ‘CAS‐Canglong’. The acronym of CAS stands for the Chinese Academy of Sciences; Canglong is a figure from ancient Chinese mythology, believed to control rainfall.

This model is explicitly designed to capture the complexities of sub‐seasonal to seasonal scale forecasting by incorporating both spatial and temporal dimensions. We incorporated time as the third dimension to consider predictors over multiple time steps. The model operates on an encoder‐decoder framework utilizing the Transformer strategy. By employing the Swin‐Transformer architecture, it deeply extracts correlation information embedded in the predictors that is pertinent to land‐atmosphere interactions. The data are processed through multi‐head self‐attention (MSA) and window multi‐head self‐attention modules (W‐MSA), which upscale and restore the information to the output layer, enhancing prediction accuracy and detail.

Specifically, this model is designed to handle normalized multivariable data in the format *T_in_ × C × N_lat_ × N_lon_*, where *T_in_* represents the number of consecutive months used as input predictors (set to 16 months), *C* is the number of features (set to 8 corresponding to the predictor variables), and *N_lat_* × *N_lon_* are the latitude and longitude grid points (721 × 1440, respectively). Therefore, the total input dimension is 16 × 8 × 721 × 1440. The model outputs predictions for the subsequent months, with adjustments for various forecasting needs. Typically, predictions are made for the following three months with dimensions *T_out_ × N_lat_ × N_lon_ =* 3 × 721 ×1440, which is a standard setup for sub‐seasonal scale predictions. The Tout parameter can be adjusted according to different prediction objectives, ranging from one to three, and up to a maximum of 16 months.

As shown in Figure S24, the data undergo normalization and preprocessing before being passed through the W‐MSA and SW‐MSA processes, during which they are downscaled to extract features, inspired by the successful applications of Pangu‐Weather and Fuxi‐Weather models [29, 31]. We employed a patch embedding layer with dimensions of 2 × 4 × 4, which is similar to that of the Vision Transformer. After this block embedding layer, the data are fed into the Swin Transformer, which utilizes an encoder‐decoder architecture. To further tailor the model for Earth system applications, we implemented an Earth‐specific positional bias strategy, which adapts techniques from computer vision tasks to Earth system imagery. This adaptation helps enhance the model's relevance and effectiveness in interpreting complex geographical and atmospheric data.

### 4.3 Training Approach

CAS‐Canglong processes batches of data (batch size B = 4), each containing a 16‐month consecutive sequence and 8 features as multi‐dimensional input. It predicts SSTs for several subsequent months as targeted outputs to train the model. Unlike other models that may use a rolling prediction strategy, this model focuses exclusively on high‐precision SST simulations, intentionally disregarding other variables. Furthermore, it has the capability to dynamically adjust and output forecasts for up to 16 months in advance, accommodating a range of forecasting needs while maintaining a focus on accuracy. The period from January 1959 to August 2009 was used for training, from September 2009 to April 2016 for validation, and from May 2016 to December 2022 for testing. The ratio of the training, validation, and testing sets is approximately 8:1:1.

The temporal period selection was carefully considered. All evaluations were conducted using the test period dataset (May 2016 to December 2022) to avoid information leakage. When comparing with other models, we selected the overlapping period for comparison. For instance, we selected the data starting from 2010 for NMME comparison, and the data from 2015 for ECMWF S2S comparison.

To train the model efficiently and enhance prediction accuracy, we employed a combination of several loss functions. The primary metric utilized is the Mean Squared Error (MSE), which evaluates the model's ability to accurately interpret SST data across a three‐month observational period. This approach is critical in accelerating the model's convergence, as it facilitates a rapid and effective comprehension of the SST data, thereby enhancing the overall performance of the forecasting system:

(1) $$\;L_{fmse}=\frac{1}{T_{out}}\;\sum_{t=1}^{T_{out}}{\left(X_{t,l}-{\hat{X}}_{t,l}\right)}^{2}$$

In this context, *X*_*t*,*l*_ and ${\hat{X}}_{t,l}$ represent the actual and predicted values of the SST for the month t + l, respectively. The loss function *L_fmse_* quantifies the model's capability to forecast up to *T_out_* months lead time, and is a commonly utilized metric for temperature prediction. Using absolute SST values during training may unfairly penalize the model due to potential climatology biases. To address this issue and enhance the model's predictive capability, we introduce a new loss function, *L_SSTA_*, which specifically focuses on the prediction of SSTA

(2) $$L_{SSTA}=\frac{1}{T_{out}}\;\sum_{t=1}^{T_{out}}{\left(\left(X_{t,l}-\bar{X_{l}}\right)-\left({\hat{X}}_{t,l}-\bar{X_{l}}\right)\right)}^{2}$$

Here, $\bar{X_{l}}$ represents the climatological multi‐year mean SST for the *l*‐th month. This loss function ensures that the model explicitly learns to predict deviations from the climatological mean, thereby mitigating the impact of climatology biases. While MSE is effective in enhancing convergence by focusing on the model's ability to interpret SST data during the observation period, it may lead to a prioritization of short‐term forecast accuracy over long‐term precision. To address this potential imbalance, we further introduced a second loss function designed to distribute penalization more evenly across the entire forecast period. This strategic approach ensures a balanced focus on both immediate and extended forecast accuracy:

(3) $$\begin{array}{ccc} & & L_{corr}=\\ & & \frac{1}{T_{out}}\;\sum_{l=2}^{T_{\mathrm{out}}}argmax\left\{0,0.5\;-\;\frac{\sum_{t=1}^{T}\left(X_{t,l}-\bar{X_{l}}\right)\;\left({\hat{X}}_{t,l}-\bar{\hat{X_{l}}}\right)}{\sqrt{\sum_{t=1}^{T}{\left(X_{t,l}\;-\;\bar{X_{l}}\right)}^{2}}\sqrt{\sum_{t=1}^{T}{\left({\hat{X}}_{t,l}\;-\;\bar{\hat{X_{l}}}\right)}^{2}}}\right\} \end{array}$$

where $\bar{X_{l}}$ and $\bar{\hat{X_{l}}}$ represent the mean observed and predicted values of sea surface temperature for the 𝑙‐th month following the observation period, respectively. The loss function *L_corr_* specifically penalizes instances where the Pearson correlation coefficient between the actual and predicted SST values falls below 0.5 in long‐term forecasts. This design underscores the critical importance of maintaining accuracy in long‐term predictions, ensuring that the model not only predicts accurately in the short term but also retains reliability over extended periods. The combined loss function, defined as the sum of *L_fmse_*, LSSTA, and *L_corr_*, provides a comprehensive approach to model training, balancing the focus between immediate accuracy and long‐term predictive reliability.

The Adam optimization algorithm is employed to optimize the model, starting with an initial learning rate of 1.0 × 10^−4^ and using a cosine decay strategy for learning rate adjustment. This strategy gradually reduces the learning rate, aiding in fine‐tuning the model's weights as training progresses. The model is initially trained on four A100 GPUs, each with 80GB of memory, which provides substantial computational power for handling the intensive calculations required for SST predictions. Additionally, the model is designed to support transfer learning, allowing for its adaptation to different SST regions. Figure S25 evaluate the performance of CAS‐Canglong with CMIP6 transfer learning. This flexibility enhances its utility across various geographic settings by fine‐tuning the pre‐trained model with data specific to each region. To further reduce prediction uncertainty, several identical models are trained using the same methodology but with different initialization parameters. This diversity in initial conditions enables ensemble predictions, where the outputs of multiple models are aggregated to produce a single forecast. This ensemble approach strongly enhances the reliability and accuracy of the forecasts by averaging out individual model biases and variances, providing a more robust prediction.

### 4.4 SST Regions and Related Climate Indices

Several observed SST and related indices across different regions of the world were used for model assessment: El Niño Southern Oscillation, Arctic Oscillation, Southern Oscillation. These regions are important for S2S forecasting. For instance, the SSTs in the South Atlantic, South Pacific, and Arctic play a crucial role in forecasting East Asian summer monsoon rainfall. These SSTs not only serve as key predictors but also enhance the timeliness of these forecasts, enabling predictions to be made up to three months in advance.

The Niño 3.4 region is widely used to define the ENSO, a naturally occurring oceanic phenomenon involving atmospheric coupling in the tropical Pacific Ocean. Niño 3.4 index is based on the five‐month rolling mean of the gridded SST in the Niño 3.4 region (170°W‐120°W, 5°S‐5°N). The two extreme phases of ENSO are determined by the SST anomaly persisting above or below 0.5°C for more than five months. El Niño is identified when the SST anomaly is above 0.5°C for more than five months, and La Niña is identified when the SST anomaly is below 0.5°C for more than five months. In this context, we examine the SSTA value in Niño 3.4 region.

The Arctic Oscillation (AO) is a large‐scale mode of climate variability. It is used to measure the 1000 mb height pressure poleward of 20°N. We assess the SST in this region.

The Indo‐Pacific Warm Pool (35°E‐75°W, 15°S‐15°N) is characterized by its SST consistently exceeding 28°C. This vast region represents the largest reservoir of warm water in the global oceans and plays a crucial role in regulating global climate patterns.

The Southern Ocean (SO) is a dominant part of the global thermohaline circulation, playing a key role in climate variability and change. We adopted the SO definition according to Luo et al., which are divided into three regions: SIO (20°E‐160°E, 40°S‐60°S), SPO (160°E‐70°W, 40°S‐60°S), and SAO (70°W‐20°E, 40°S‐60°S).

### 4.5 Evaluation Metrics

We first define the SSTA. For both ERA5 reanalysis data and model predictions, including GCM and CAS‐Canglong, the SSTA is calculated as the deviation of SST from its own climatological mean for each corresponding calendar month:

(4) $$SST\;A_{i,j,l}=SST_{i,j,l}-{\bar{\mathrm{SST}}}_{i,j,clim\left(l\right)}$$

where *SST_i,j,l_* is the SST at longitude *i*, latitude *j*, and time *l*. ${\bar{SST}}_{i,j,clim(l)}$ is the climatological mean SST at the same location and calendar month. For model predictions, the climatology is calculated from the model output itself; for ERA5, the climatology is based on the ERA5 dataset.

For evaluating spatial performance, we assessed two metrics, ARMSE and correlation ACC, defined as follows:

(5) $$ARMSE=\sqrt{\frac{\sum_{i=1}^{N_{lon}}\sum_{j=1}^{N_{lat}}W\left(i\right){\left({\hat{X}}_{i,j,l}-X_{i,j,l}\right)}^{2}}{N_{lon}\times N_{lat}}}$$

(6) $$ACC=\sqrt{\frac{\sum_{i=1}^{N_{lon}}\sum_{j=1}^{N_{lat}}W\left(i\right){\hat{X}}_{i,j,l}X_{i,j,l}}{\sum_{i=1}^{N_{lon}}\sum_{j=1}^{N_{lat}}W\left(i\right){\left({\hat{X}}_{i,j,l}\right)}^{2}\times \sum_{i=1}^{N_{lon}}\sum_{j=1}^{N_{lat}}W\left(i\right){\left(X_{i,j,l}\right)}^{2}}}$$

where *X*_*i*,*j*, *l*_ and ${\hat{X}}_{i,j,l}$ represent the ERA5 ground truth and predicted values of SSTA on a given grid cell (longitude *i*, latitude *j*) for the 𝑙 time month, respectively.

For evaluating temporal performance, we used the metric of Temporal Correlation Coefficient (TCC). The first step is to form the area‐weighted series:

(7) $$x_{t,l}=\frac{\sum_{i,j\in R}W\left(i\right)X_{i,j,t,l}}{\sum_{i,j\in R}W\left(i\right)}$$

(8) $${\hat{x}}_{t,l}=\frac{\sum_{i,j\in R}W\left(i\right){\hat{X}}_{i,j,t,l}}{\sum_{i,j\in R}W\left(i\right)}\;$$

*X*_*t*,*l*_ and ${\hat{X}}_{t,l}$ are the observed and predicted regional‐index (or single‐grid‐point) time series at lead *l*. ${\bar{x}}_{l}$ is the time mean over the verification dates. R is the index region (e.g. always Niño3.4 region). W(i) represents the area weighted formula of Earth, defined as follows:

(9) $$W\;\left(i\right)=N_{lat}\;\times \frac{cos\phi_{i}}{\sum_{i=1}^{N_{lat}}cos\phi_{i}}$$

where ϕ_*i*_ represents the latitude angle. Then, we quantify TCC for individual grid cells as follows:

(10) $$TCC\;\left(l\right)=\frac{\sum_{t=1}^{T}\left({\hat{x}}_{t,l}-{\bar{\hat{x}}}_{l}\right)\left(x_{t,l}-{\bar{x}}_{l}\right)}{\sqrt{\sum_{t=1}^{T}{\left({\hat{x}}_{t,l}-{\bar{\hat{x}}}_{l}\right)}^{2}}\sqrt{\sum_{t=1}^{T}{\left(x_{t,l}-{\bar{x}}_{l}\right)}^{2}}}$$

To characterize the intrinsic memory of observed climate modes, we calculate the sample autocorrelation *AR_l_* of the observed index anomalies as a function of lag *l*:

(11) $$A\;R_{l}=Corr\left(y_{t},y_{t+1}\right)$$

where *y_t_* and *y_t+l_* denote the observed index anomalies at time *t* and *l* months later, respectively, and *l* is the lag in months. For the special case of *l =* 1, *AR_1_* is equivalent to the correlation skill of the persistence baseline. In our evaluations, *AR_1_* and the persistence baseline serve as essential benchmarks, where *AR_1_* is reported alongside climate index time series in the main text (e.g., Figure 4), while the persistence baseline provides a lead‐dependent benchmark across forecast horizons (Figure 1g).

The ARMSE and ACC values were averaged over all times and weighted pixels. These two metrics are used to make comparison and evaluating prediction skills from different models in specific regions. In parallel, the TCC is widely employed to assess phase consistency and temporal coherence over extended forecast horizons, such as for the Niño‐3.4 anomaly series across leads 1–12 months in Figure S11. To further benchmark model performance, an autoregressive baseline is evaluated in Figure S17, while probabilistic ensemble calibration and spatial scale fidelity are also included, more details on these metrics and the corresponding results can be found in Figures S17–S18. For evaluating CAS‐Canglong performance, we define the improvement as the ARMSE reduction percentage from multi‐model ensemble average to CAS‐Canglong prediction.

## Author Contributions

**Longhao Wang**: methodology, software, data curation, investigation, validation, formal analysis, visualization, Writing – original draft. **Xuanze Zhang**: methodology, data curation, validation, formal analysis, supervision, writing – original draft, writing – review and editing, funding acquisition, resources. **Ruby L. Leung**: writing – review and editing. **Francis H. S. Chiew**: writing – review and editing. **Amir AghaKouchak**: writing – review and editing. **Kairan Ying**: writing – review and editing. **Yongqiang Zhang**: methodology, writing – review and editing, writing – original draft, conceptualization, visualization, supervision.

## Funding

Yongqiang Zhang was supported by the National Key R&D Program of China (Grant No. 2022YFC3002804) and the Inner Mongolia Autonomous Region Water Conservancy Research Special Project (Grant No. NSK202301) and the Talent Program of the Ministry of Science and Technology of China. Xuanze Zhang thanks the support from the Youth Innovation Promotion Association CAS (2022053) and the “Kezhen‐Bingwei” Youth Talents Project from IGSNRR CAS (2021RC003). Ruby L. Leung was supported by the Office of Science, U.S. Department of Energy Biological and Environmental Research as part of the Global and Regional Model Analysis program area. Pacific Northwest National Laboratory is operated by Battelle for the U.S. Department of Energy under Contract DE‐AC05‐76RL01830.

## Conflicts of Interest

The authors declare no conflicts of interest.

## Supporting information




**Supporting File**: advs78271‐sup‐0001‐SuppMat.docx.

## Data Availability

For training and testing, we downloaded a subset of the ERA5 dataset of 63 years from https://cds.climate.copernicus.eu/. Data used and produced in this research can be download from Figshare repository: http://doi.org/10.6084/m9.figshare.26779969. The real‐time operational forecast products generated by this model are available at https://canglong.earth/sst/. The code and implementation details for the CAS‐Canglong model data are publicly accessible at https://github.com/GISWLH/CAS‐Canglong/.
