# Supplementary material for: Improved Skills for Predicting Sub‐Seasonal to Seasonal Global Sea Surface Temperature
Source: Adv Sci (Weinh). 2026 Oct 15:e78271. Online ahead of print. doi: 10.1002/advs.78271 (PMC13672690; doi:10.1002/advs.78271)
Supplement: Supplementary file 1 [file ADVS-9999-e78271-s001.docx]

Supporting Information

Improved skills for predicting sub-seasonal to seasonal

global sea surface temperature

Longhao Wang^#^, Xuanze Zhang^#^, Ruby L. Leung, Francis H.S. Chiew, Amir AghaKouchak, Kairan Ying, and Yongqiang Zhang*

L. Wang, X. Zhang, Y. Zhang

Key Laboratory of Water Cycle and Related Land Surface Processes, Institute of Geographic Sciences and Natural Resources Research, Chinese Academy of Sciences

Beijing 100101, China
E-mail: zhangyq@igsnrr.ac.cn

L. Wang

College of Resources and Environment
University of Chinese Academy of Sciences

Beijing 100049, China

R. L. Leung

Pacific Northwest National Laboratory

Richland, Washington 99352, USA

F. H.S. Chiew

CSIRO Environment, Black Mountain

Canberra, ACT 2601, Australia

A. AghaKouchak

Center for Hydrometeorology and Remote Sensing, University of California, Irvine

California, USA

K. Ying

National Institute of Natural Hazards, Ministry of Emergency Management of China

Beijing, China

^#^Longhao Wang and Xuanze Zhang contributed equally to this work.

Table S1 summarizes recent advanced AI weather model at medium and S2S scale.

Table S2 summarizes the design of sensitivity experiments.

Table S3 summarizes the full names and abbreviations of the input variables used in the CAS-Canglong, 3D-Geoformer, and ENSO-ASC.

Figure S1 summarizes the mean ARMSE of the CAS-Canglong and NWP S2S models compared with ERA5 ground truth for global SST predictions for the period from 2016 to 2020.

Figure S2 assesses the predictive skills for the CAS-Canglong and the state-of-the art GCM S2S models by comparing with the Hadley Centre Sea Surface Temperature data set (HadSST).

Figure S3 shows our prediction for the 2015-2022 El Niño and La Niña events, compared with ECMWF prediction.

Figure S4 compares spatial patterns of SSTA between CAS-Canglong predictions and observations.

Figure S5 shows the heat map of correlation skill in the CAS-Canglong for the Niño 3.4 index.

Figures S6 shows Hovmöller diagrams of latitude-averaged SSTA across test period.

Figures S7 shows the Global marine heatwave (MHW) months from ERA5 and 1-month lead predictions using CAS-Canglong.

Figure S8 shows the MHW events predictive skill in the Tasman Sea.

Figure S9 shows the spatial distribution of MHW months during 2016/2017 over the Tasman Sea near New Zealand.

Figure S10 provides the prediction of the Warm Pool Area in the Indo-Pacific.

Figure S11 shows the Niño-3.4 index anomaly correlation coefficient (ACC) as a function of forecast lead.

Figure S12 shows spatial pattern correlation coefficient of SSTA forecasts as a function of forecast lead.

Figure S13 shows area-weighted root mean square error (ARMSE) of sea-surface temperature anomaly forecasts as a function of forecast lead.

Figure S14 shows the time series of SSTA predictions in major regions for the period 2016-2022.

Figure S15 shows the comparison with three AI models: FuXi-S2S, 3D-CNN, and 3D-GeoFormer.

Figure S16 shows the global weighted ARMSE using 3D-CNN as a baseline.

Figure S17 summarizes the ARIMA’s performance as a baseline model.

Figure S18 shows the ranking of Fractional Skill Score (FSS) with numerical and deep learning model.

Figure S19 shows the ensemble forecast skill scores for CAS-Canglong compared to traditional numerical models and recent deep learning models. Specially for Continuous Ranked Probability Score (CRPS) and Spread-Skill Ratio (SSR).

Figure S20 presents rank histograms show that CAS-Canglong’s ensembles are well calibrated.

Figure S21 shows conceptual mechanisms underlying regional SST forecast difficulty in the Arctic Ocean and southern Indian Ocean.Figure S22 shows Power Spectral Density plots compared to ERA5.

Figure S23 shows the SHAP results in relative contributions of all time lags to SST predictions.

Figure S24 displays the architecture of CAS-Canglong.

Figure S25 evaluate the performance of CAS-Canglong with CMIP6 pre-training. It shows the prediction ability of global SST and ENSO.

**Fig. S1 | ARMSE of the CAS-Canglong and NWP S2S models compared with ERA5 ground truth.** All predictions were made one month in advance for global SST from 2015 to 2020. **a**, SST prediction in one month advance. **b**, SST prediction in two months advance. **c**, SST prediction in three months advance. The error bars represent the mean error ± one standard deviation. Anomalies are calculated based on each model’s own climatology. The persistence baseline assumes that the SSTA at a given time remains unchanged for all future lead times.

**Fig. S2 |** **Predictive skills for the CAS-Canglong and the state-of-the art GCM S2S models, evaluated against the Hadley Centre Sea Surface Temperature data set (HadSST).** Left panels are for correlation (R) and right panels are for RMSE over the overlapped period (from 2015 to 2020). The one to three-month lead predictions of SST from CAS-Canglong are compared to those from 22 state-of-the-art NWP models. The boxplot shows the R and RMSE distribution, generated by comparing GCM S2S modelled SST with HadSST. Each panel shows the lower whisker (Q1-1.5IQR), lower quartile (Q1), median, upper quartile (Q3), and upper whisker (Q3+1.5IQR), where the IQR = Q3-Q1. The red dots represent CAS-Canglong performance.

**Fig. S3 | Our prediction for the 2015-2022 El Niño and La Niña events compared with ECMWF.** ERA5 analyzed ground truth (black line) and model predictions (light blue line) for SST anomalies in the Niño 3.4 region (5°N-5°S, 120°W-170°W). Shades around the lines represent ± one standard deviation.

**Fig. S4 | Spatial pattern comparison of SSTA between predictions and observations. b, d, f, h**, The prediction from CAS-Canglong. **a, c, e, g**, The ground truth from ERA5. Two El Niño and La Niña events were selected in the test period for comparison. All predictions were made with a one-month lead time.

**Fig. S5 | Heat map of correlation skill in the CAS-Canglong for the Niño 3.4 index.** The figure shows the correlation skill for different forecast start months over the validation period. Darker colors indicate better predictive performance.

**Fig. S6. Hovmöller diagrams of latitude-averaged SSTA across test period.** (a) ERA5 ground truth. (c) CAS-Canglong prediction.

We have extended our analysis to include Marine Heatwave (MHW) metrics, following the definition provided by Jacox et al. (2022). Unlike daily MHW assessments, our analysis focuses on monthly S2S prediction scales. MHW thresholds specific to each month were calculated as the 90th percentile of observed SST anomalies within a 3-month rolling window. Using this definition, we compared the number of MHW months identified in the ERA5 dataset with the 1-month lead predictions from CAS-Canglong. Fig. S15 demonstrates the observed and predicted MHW months globally.

**Fig. S7 | Global marine heatwave (MHW) months from ERA5 and 1-month lead predictions using CAS-Canglong. a**, ERA5 ground truth. **b**, Predicted MHW months. The evaluation is based on the test data period from 2016 to 2022.

We also examined MHW occurrences in the Tasman Sea, located north of New Zealand and east of Australia, within the region [145°E–180°E, 20°S–50°S]. The analysis revealed that the southwestern Tasman Sea experienced severe MHW events from 2016 to 2022. Our AI model was able to largely reproduce the spatial distribution of these events.

**Fig. S8 | Marine heatwave events in the Tasman Sea: a**, The location of the Tasman Sea, north of New Zealand and east of Australia. **b**, Observed MHW months from 2016 to 2022. **c**, Predicted MHW months with a 1-month lead time using CAS-Canglong.

**Fig. S9 | MHW months during 2016/2017 over the Tasman Sea near New Zealand. a**, The observed MHW months calculated from ERA5 during 2016–2017. **b**, **c**, **d**, Predicted MHW months at lead times of 1 month, 2 months, and 3 months.

**Fig. S10 | Prediction of Warm Pool Area in the Indo-Pacific.** This area is characterized by warm SSTs exceeding 28°C. **a**, The ground truth of ERA5 for the multi-year mean warm pool area during the test period. **b**, Predictions made one months in advance. **c**, Predictions made three months in advance. In both scenarios, the warming pool boundary contour lines are rendered smoothly to highlight CAS-Canglong's accuracy in capturing the extent of the warm pool.

**Fig. S11 | Niño-3.4 index anomaly correlation coefficient (ACC) as a function of forecast lead.** Curves show CAS-Canglong, persistence, a multiple linear regression (MLR) baseline, Geoformer, FuXi-S2S, and the available individual dynamical models for leads 1–12 months. Persistence holds the observed Niño-3.4 anomaly at the forecast origin fixed for every lead. MLR is a one-step ridge-regularized autoregression with an intercept and four monthly predictors, fitted on the first 36 months through April 2019 and recursively evaluated from May 2019.

**Fig. S12 | Spatial pattern correlation coefficient of SSTA forecasts as a function of forecast lead.** Forecasts are evaluated for leads 1–12 months over the common May 2016–December 2022 verification period, with forecasts and observations paired by valid month. Forecast and observed SST fields are converted to SSTA using their respective calendar-month climatology; ocean grid cells within each region are flattened and correlated using Pearson's r, and the plotted curves show the mean PCC over valid months.

**Fig. S13 | Area-weighted root mean square error (ARMSE) of sea-surface temperature anomaly forecasts as a function of forecast lead.** Forecasts are evaluated for leads 1–12 months over the common May 2016–December 2022 verification period, with each forecast lead aligned to the corresponding valid month. Forecast and observed SST fields use their respective calendar-month climatologies to form SSTA; regional ARMSE is calculated as the cosine-latitude-weighted RMSE over ocean grid cells and averaged over valid months.

**Fig. S14 | Time series of SSTA predictions in major regions for the period 2016-2022.** Predictions are conducted every month from 2016 to 2022, and the results are merged to generate a single time series. The black line represents the ERA5 ground truth, while the blue lines show the model predictions for one to three months lead time, with colors ranging from dark to light blue corresponding to one, two, and three months, respectively. **a**, AO. **b**, SAO. **c**, SPO. **d**, SIO.

We include comparisons with three AI models: FuXi-S2S (Chen et al., 2024), 3D-CNN (Ham et al., 2019) and 3D-GeoFormer (Zhou et al., 2023), respectively. Using the publicly available code and data provided in these studies, we reproduced the models and download the dataset to ensure fair and consistent comparisons. For this purpose, we used the same input variables and temporal dimensions as those employed in CAS-Canglong. For all four AI models, including CAS-Canglong, we used identical training, validation, and testing periods (training: 1959.1 to 2009.8; validation: 2009.9 to 2016.4; testing: 2016.5 to 2022.12) and the same input data to ensure fair evaluations. However, since FuXi-S2S only provides data up to 2021, its performance in 2022 was not evaluated. Table S1 summarizes recent AI weather model which have potential ability to S2S predictions. Figure. S9 shows the comparison with three AI models. Firstly, for the 3D-CNN and 3D-Geoformer models, the original authors provided the model codes. We reproduced their model structures without modifying the parameters and used the same training period and variables as CAS-Canglong to ensure a fair comparison. For the Fuxi-S2S model, the authors did not provide the original model code but made hindcast data available, whose available for evaluation is limited to 2017–2021. (https://huggingface.co/datasets/FudanFuXi/FuXi-S2S). Therefore, we compared CAS-Canglong with the Hindcast results provided by Fuxi-S2S. We utilized the hindcast results from FuXi-S2S, which include 51 ensemble members. To ensure computational efficiency and comparability, we generated 20 ensemble members for CAS-Canglong and selected the first 20 members from FuXi-S2S for comparison.

**Fig. S15 | Comparison with three AI models: FuXi-S2S, 3D-CNN, and 3D-GeoFormer. a**, The SSTA of Nino 3.4 region. **b**, Overall mean ARMSE values in all regions. The 3D-GeoFormer and 3D-CNN models were trained using the same period and data as CAS-Canglong, while the FuXi-S2S results are based on their provided hindcast data.

**Fig. S16 | Global weighted anomaly ARMSE.** CAS-Canglong compared with traditional numerical models and recent deep learning models, including the 3D-CNN baseline.

**Fig. S17 | Evaluation of ARIMA’s performance as a baseline model.** **a**, Comparison of ARIMA-predicted SST with ERA5 ground truth in the Niño3.4 region. The blue line represents ARIMA predictions, the green line represents CAS-Canglong predictions, and the black line represents the ERA5 ground truth. The background shading indicates the Niño3.4 index. **b**, ACC of all predictions for one-month lead global SST from 2016 to 2020. ARIMA is highlighted in red as the baseline model.

We incorporated additional verification metrics from WeatherBench2, including the Fractions Skill Score (FSS), Continuous Ranked Probability Score (CRPS), and spread-skill ratio (SSR). The FSS is formulated as:

$$FSS\left( n \right)=1-\frac{MSE(n)}{MSE_{ref}(n)}$$

where *MSE(n)* represents the mean squared error between the binary fields of predictions and observations after spatial smoothing with a window size n, and *MSE_ref(n)* denotes the sum of mean squared differences between each field and its mean value. These terms are explicitly defined as:

$$MSE\left( n \right)=\frac{\sum_{i=1}^{N_{lon}} \sum_{j=1}^{N_{lat}} W\left( i \right)\left( \bar{O}_{i,j,l}\left( n \right)-\bar{O}\left( n \right) \right)}{\sum_{i=1}^{N_{lon}} \sum_{j=1}^{N_{lat}} W\left( i \right)}$$

$$MSE_{ref}\left( n \right)=\frac{\sum_{i=1}^{N_{lon}} \sum_{j=1}^{N_{lat}} W\left( i \right)\left( \bar{O}_{i,j,l}\left( n \right)-\bar{O}\left( n \right) \right)^{2}}{\sum_{i=1}^{N_{lon}} \sum_{j=1}^{N_{lat}} W\left( i \right)}+\frac{\sum_{i=1}^{N_{lon}} \sum_{j=1}^{N_{lat}} W\left( i \right)\left( \bar{P}_{i,j,l}\left( n \right)-\bar{P}\left( n \right) \right)^{2}}{\sum_{i=1}^{N_{lon}} \sum_{j=1}^{N_{lat}} W\left( i \right)}$$

Here, $\bar{O}_{i,j,l}\left( n \right)$ represents the spatially smoothed observational value at position *(i,j)* and time *l* using an *n×n* window, while$\bar{O}\left( n \right)$ denotes the spatial mean of the entire observational field. $\bar{P}_{i,j,l}\left( n \right)$ and $\bar{P}\left( n \right)$ represent the smoothed predicted values and their spatial mean, respectively. In our implementation, we set n=16 (approximately corresponding to a 4° lat-lon grid). The FSS ranges from 0 to 1, with higher values indicating better skill (Faggian et al., 2015). The FSS results are shown in Fig. S13. The CRPS is a widely adopted metric for evaluating ensemble forecast quality. For a continuous variable, the CRPS is mathematically defined as:

$$CRPS=\int_{-\infty}^{\infty} {(F\left( x \right)-H\left( x-y \right))}^{2}dx$$

where *F(x)* denotes the forecast's cumulative distribution function (CDF), H(x-y) represents the Heaviside step function, and y is the observed value. For an ensemble forecast system with M members, the CRPS at a specific spatiotemporal point (i,j,l) is computed as:

$$CRPS_{i,j,l}=\frac{1}{M}\sum_{m=1}^{M} \left| \hat{X}_{i,j,l,m}-X_{i,j,l} \right|-\frac{1}{2M^{2}}\sum_{m=1}^{M} \sum_{n=1}^{M} \left| \hat{X}_{i,j,l,m}-X_{i,j,l,n} \right|$$

where $\hat{X}_{i,j,l,m}$represents the forecast value from the m-th ensemble member. i, j, and l denotes the spatial-temporal coordinates (longitude, latitude, time). $X_{i,j,l}$is the corresponding observed value. M is the total number of ensemble members, here we calculated 20 members. The CRPS values were calculated for both CAS-Canglong and multi-model ensembles for comparative analysis. The metric ranges from 0 to ∞, with lower values indicating superior probabilistic forecasting skill (Rasp et al., 2024). The CRPS results are shown in Fig. S14

SSR provides insights into the reliability and consistency of ensemble forecasts. SSR relates the spread (variability) of ensemble members to the skill (accuracy) of the ensemble mean forecast. A well-calibrated ensemble system should exhibit an SSR close to 1. The SSR is obtained by dividing ‘spread’ by RMSE with spread being:

$$Spread\left( v,t \right)=\sqrt{\frac{\sum_{i=1}^{N_{lon}} \sum_{j=1}^{N_{lat}} W(i)\cdot var(\hat{X}_{i,j,l})}{N_{lat}\times N_{lon}}}$$

Here, *var (·)* indicates the variance in the ensemble dimension. The spread and RMSE values averaged over all forecasts are used for computing SSR. A perfectly reliable ensemble reports an SSR of 1.0. The SSR results are presented in Fig. S14.

**Fig. S18 | The ranking of Fractional Skill Score (FSS).** Comparisons are made between CAS-Canglong, traditional numerical models, and recent deep learning models.

**Fig. S19 | Ensemble forecast skill scores for CAS-Canglong compared to traditional numerical models and recent deep learning models. a,** Continuous Ranked Probability Score (CRPS). **b**, Spread-Skill Ratio (SSR).

**Fig. S20. Rank histograms show that CAS-Canglong’s ensembles are well calibrated.** CAS-Canglong generally has flat rank histograms, in many cases flatter than those of Cansips, and in almost all cases substantially flatter than Fuxi-S2S. These plots show rank histograms of CAS-Canglong, Cansips, and Fuxi-S2S at different lead times Nino, AO, WP, SIO, SPO, and SAO regions.

**Fig. S21 | Conceptual mechanisms underlying regional SST forecast difficulty in the Arctic Ocean and southern Indian Ocean.** Panel (a) shows the moving marginal-ice-zone boundary, atmospheric boundary-layer contrast, air-sea moisture exchange, and mixed-layer variability in the Arctic Ocean. Panel (b) shows Southern Annular Mode-related wind variability, air-sea heat exchange, ocean memory, bathymetric steering of Antarctic Circumpolar Current fronts, and eddy-rich meandering transport in the southern Indian Ocean.

**Fig. S22. Power Spectral Density (PSD) plots.** **a**, Full spectrum result comparing CAS-Canglong predictions with ERA5 observations. **b**, The zoomed regions.

**Fig. S23 | The relative contributions of all time lags to SST predictions.** Results were obtained using SHAP to interpret the deep learning model. The SHAP analysis is based on all global ocean pixels. Bars represent the mean importance of each lag variable, dots indicate individual effects, and error bars show the mean ± standard deviation.

**Fig. S24 | Architecture of CAS-Canglong. a**, Includes a data preprocessing and output recovery module. The core of the model is the Swin-Transformer. The downscaled data enter the Swin-Transformer module, where features and relationships from the ocean-atmosphere system data are extracted through the MSA and W-MSA modules. **b,** The schematic diagram illustrates the Swin-Transformer adapted for Earth surface tasks, with a progressively expanding receptive field to extract relationships within and between adjacent Earth pixels. **c**, The Transformer module based on the encoder-decoder structure, highlighting the specific roles of MSA, followed by a linear output layer at the end of the decoder to finalize the prediction output. The "Masked multi-head 3D attention" in Swin-Transformer extends the standard attention mechanism into three dimensions. The model could effectively capture spatiotemporal dependencies by dividing the input into non-overlapping 3D blocks and performing self-attention within each block.

**Fig. S25 | Evaluation the performance of CAS-Canglong with CMIP6 pre-training. a**, The model predictions with ERA5 ground truth in the Niño3.4 region. **b**, The ARMSE of all predictions for one-month lead global SST during the period 2016–2020 is shown. The green line represents CAS-Canglong-CMIP6 predictions, the orange line represents CAS-Canglong predictions, and the black line represents the ERA5 ground truth. The background shading indicates the Niño3.4 index.

**Table S1. Recent advanced AI weather model at medium and S2S scale**

| Model | Source | Method | Type | Scale | Resolution | Reference |
| --- | --- | --- | --- | --- | --- | --- |
| Pangu | Huawei | 3D-Transformer | Deterministic | Weather | 0.25° | Bi et al., 2023 |
| GraphCast | Google | Graph neural network | Deterministic | Weather | 0.25° | Lam et al., 2023 |
| FuXi | Fudan University | U-Transformer | Deterministic | Weather | 0.25° | Chen et al., 2023 |
| NeuralGCM | Google | Hybrid | Deterministic/Ensemble | Weather | 0.7° & 1.4° | Kochkov et al., 2024 |
| GenCast | Google | Diffusion | Ensemble | Weather | 0.25° | Price et al., 2024 |
| FuXi-S2S | Fudan University | VAE- Transformer | Deterministic | S2S | 0.25° | Chen et al., 2024 |

**Table S2.** Design of sensitivity experiments.

|  | Ocean variable | Atmosphere | Energy |
| --- | --- | --- | --- |
| S0 baseline | Climatology | Time-variable | Time-variable |
| S1 Atmosphere | Time-variable | Climatology | Time-variable |
| S2 Energy | Time-variable | Time-variable | Climatology |
| S3 Final model | Time-variable | Time-variable | Time-variable |

**Table S3. Full names and abbreviations of the input variables used in the CAS-Canglong, 3D-Geoformer, and ENSO-ASC.**

| Model name | Full name | Abbreviation |
| --- | --- | --- |
| CAS-Canglong | Sea surface temperature | SST |
|  | Mean sea level pressure | MSL |
|  | 10m U-component of Wind | U10 |
|  | 10m V-component of Wind | V10 |
|  | Geopotential height at 500 hPa | H500pa |
|  | Geopotential height at 850 hPa | H850pa |
|  | Surface latent heat flux | SLHF |
|  | Net solar radiation | NSR |
| 3D-Geoformer | seven-layer sea surface temperature anomalies (at 5, 20, 40, 60, 90, 120, and 150 m) | SSTA |
|  | sea surface zonal wind stress | tauu |
|  | sea surface meridional wind stress | tauv |
| ENSO-ASC | Sea surface temperature | SST |
|  | 10m U-component of Wind | U10 |
|  | 10m V-component of Wind | V10 |
|  | Precipitation rate | Pr |
|  | Cloud Water Content | Cldwat |
|  | Water Vapor Content | Prw |

Supplementary references:

Bi, K. F. (2023). Accurate medium-range global weather forecasting with 3D neural networks. Nature, 619, 533-. https://doi.org/10.1038/s41586-023-06185-3

Chen, L. (2023). FuXi: A cascade machine learning forecasting system for 15-day global weather forecast. Npj Climate and Atmospheric Science, 6(1). <https://doi.org/10.1038/s41612-023-00512-1>

Chen, L. (2024). A machine learning model that outperforms conventional global subseasonal forecast models. Nature Communications, 15(1). <https://doi.org/10.1038/s41467-024-50714-1>

Faggian, N., Roux, B., Steinle, P., & Ebert, B. (2015). Fast calculation of the fractions skill score. MAUSAM, 66(3), 457–466.

Gu, Xihui, Zaiming Jiang, Yansong Guan, Ming Luo, Jianfeng Li, Lunche Wang, Xiang Zhang, Dongdong Kong和Liangyi Wang. 2025. 《Frequent Land-Ocean Transboundary Migration of Tropical Heatwaves under Climate Change》. *Nature Communications* 16(1):3400. doi: [10.1038/s41467-025-58586-9](https://doi.org/10.1038/s41467-025-58586-9).

Jacox, M. G. et al. Global seasonal forecasts of marine heatwaves. Nature 604, 486–490 (2022).

Ham, Y. G., Kim, J. H., & Luo, J. J. (2019). Deep learning for multi-year ENSO forecasts. *Nature*, *573*, 568-. https://doi.org/10.1038/s41586-019-1559-7

Kochkov, D., Yuval, J., Langmore, I., Norgaard, P., Smith, J., Mooers, G., Klöwer, M., Lottes, J., Rasp, S., Düben, P., Hatfield, S., Battaglia, P., Sanchez-Gonzalez, A., Willson, M., Brenner, M. P., & Hoyer, S. (2024). Neural general circulation models for weather and climate. *Nature*, *632*(8027), 1060–1066. https://doi.org/10.1038/s41586-024-07744-y

Lam, R. (2023). Learning skillful medium-range global weather forecasting. *Science*, *382*. https://doi.org/10.1126/science.adi2336

Price, I., Sanchez-Gonzalez, A., Alet, F., Andersson, T. R., El-Kadi, A., Masters, D., Ewalds, T., Stott, J., Mohamed, S., Battaglia, P., Lam, R., & Willson, M. (2025). Probabilistic weather forecasting with machine learning. Nature, 637(8044), 84–90. <https://doi.org/10.1038/s41586-024-08252-9>

Rasp, S. *et al.* WeatherBench 2: A benchmark for the next generation of data-driven global weather models. Preprint at <https://doi.org/10.48550/arXiv.2308.15560> (2024).

Zhou, L., & Zhang, R.-H. (2023). A self-attention-based neural network for three-dimensional multivariate modeling and its skillful ENSO predictions. Science Advances, 9(10). https://doi.org/10.1126/sciadv.adf2827
